# Periodontitis, Blood Pressure, and the Risk and Control of Arterial Hypertension: Epidemiological, Clinical, and Pathophysiological Aspects—Review of the Literature and Clinical Trials

**DOI:** 10.1007/s11906-021-01140-x

**Published:** 2021-05-07

**Authors:** Stanisław Surma, Monika Romańczyk, Justyna Witalińska-Łabuzek, Maciej R. Czerniuk, Krzysztof Łabuzek, Krzysztof J. Filipiak

**Affiliations:** 1 Faculty of Medical Sciences in Katowice, Medical University of Silesia in Katowice, Medyków 18, 40-752 Katowice, Poland; 2 Specjalist Dental Practice, Jaworzno, Poland; 3 Department of Dental Surgery, Medical University of Warsaw, Warsaw, Poland; 4 Specjalist Medical Practice, Jaworzno, Poland; 5 1st Department of Cardiology, Medical University of Warsaw, Warsaw, Poland

**Keywords:** Periodontitis, Blood pressure, Arterial hypertension

## Abstract

**Purpose of Review:**

Arterial hypertension is an important risk factor for cardiovascular disease. In the world, about 45% of people suffer from arterial hypertension, while good blood pressure control is achieved by only approximately 50% of all hypertensive patients treated. The reason for the high prevalence of arterial hypertension and its poor control is low knowledge of hypertensinogenic factors. One such factor is periodontitis, which is a disease of social importance.

**Recent Findings:**

It has been shown that the occurrence of periodontitis leads to an increase in blood pressure, increasing the risk of arterial hypertension. Periodontitis can also lead to ineffectiveness of antihypertensive treatment. Some interventional studies have shown that treatment of periodontitis reduced blood pressure in patients with arterial hypertension. The pathogenesis of arterial hypertension in periodontitis is complex and concerns mainly the impairment of the vasodilatation properties of the endothelium.

**Summary:**

Hygiene and periodontitis treatment should be a method of preventing arterial hypertension and a method of increasing the effectiveness of antihypertensive treatment.

## Introduction

Arterial hypertension is one of the most common cardiovascular risk factors. In the world, about 45% of people suffer from arterial hypertension, and its incidence increases with age [1]. According to a 2014 World Health Organization (WHO) report, arterial hypertension was the cause of 51% of deaths from stroke and 45% of overall cardiovascular mortality, and it affected all age groups and ethnic groups [1]. Blood pressure remains elevated in approximately 50% of all hypertensive patients treated [2, 3]. The reasons for such a high prevalence of arterial hypertension and its poor control include, first of all, a sufficiently low social awareness of the classic factors of its occurrence and non-compliance with therapeutic recommendations [4, 5]. However, it should be emphasized that non-classical cardiovascular risk factors, including arterial hypertension, such as common periodontitis, may also contribute to an increased incidence of arterial hypertension and its poor control [6].

## Role of the Oral Microbiome in Blood Pressure Regulation

**Fig. 1:**
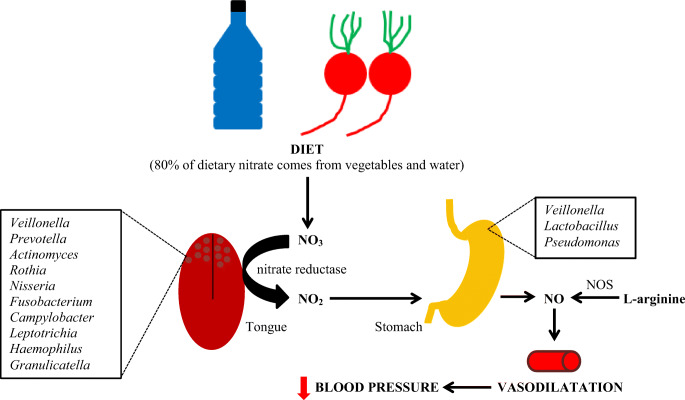
Interplay between dietary nitrate and oral commensal bacteria and blood pressure regulation. NOS, nitric oxide synthase

In normal conditions, commensal oral bacteria found in the crypts of the tongue, such as *Staphylococcus*, *Streptococcus*, *Actinomyces*, *P. melaninogenica*, *V. dispar*, *H. parainfluenzae*, *N. subflava*, *V. parvula*, *F. nucleatum* subsp. *nucleatum*, *C. concisus*, *L. buccalis*, and *P. intermedia*, are involved in the production of nitric oxide from dietary nitrates (NO_3_-NO_2_-NO reduction pathway). Due to this activity, these bacteria are an important source, in addition to endogenous synthesis, of nitric oxide (Fig. 1), which is characterized by vasodilator properties [7–9].

Nitrate/nitrite-reducing bacteria are cooperating synergistically creating an optimal oral bacterial community for NO generation. They are protective on cardiometabolic health contrary to periodontal pathogenic bacteria associated with an increased risk of adverse cardiovascular events [7].

In the study by Gordon et al., involving 446 postmenopausal women, changes in the composition of the oral microbiota depending on blood pressure were analyzed. Subgingival microbiome composition was determined using 16S rRNA sequencing with the Illumina MiSeq platform. The subjects were divided into four groups: I, with normal blood pressure; II, with elevated blood pressure/ stage I arterial hypertension; III, stage II arterial hypertension; and IV, patients using antihypertensive drugs (regardless of blood pressure). Sixty-five bacterial species demonstrated significant differences in relative abundance in women with elevated blood pressure or using hypertension medication as compared to those with normal blood pressure. After correction for multiple testing, two species, *Prevotella oral* and *Streptococcus oralis*, remained significant and were lower in abundance among women taking antihypertension medications compared to those with normal blood pressure (*p* <0.05). It was found that the composition of oral bacteria and blood pressure are correlated [10].

In summary, the oral microbiota is involved in the regulation of blood pressure. Moreover, the composition of the oral microbiota and blood pressure are correlated.

## Periodontitis

**Fig. 2:**
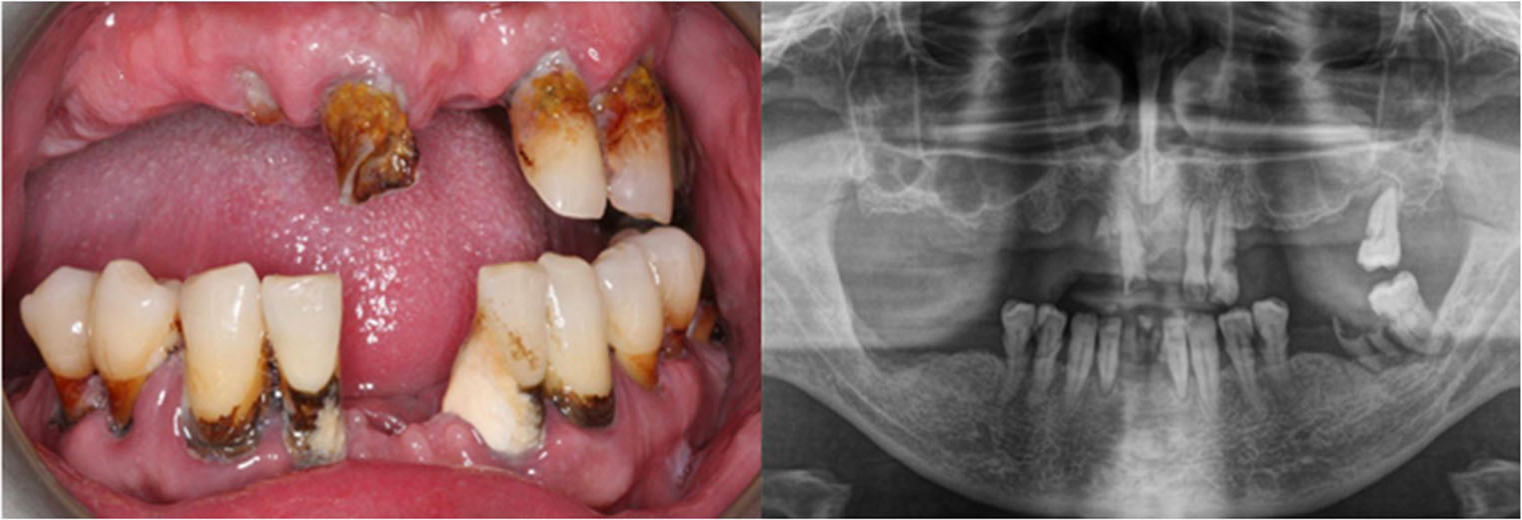
A 62-year-old man with advanced periodontal disease. Visible bone loss in the radiographic image. In the clinical picture, tartar deposits above and below the gingiva are visible

Periodontitis is a chronic, multifactorial inflammatory disease caused by dysbiotic oral microflora, causing progressive destruction of the tissues surrounding the teeth (Fig. 2) and tooth loss [11].

**Fig. 3:**
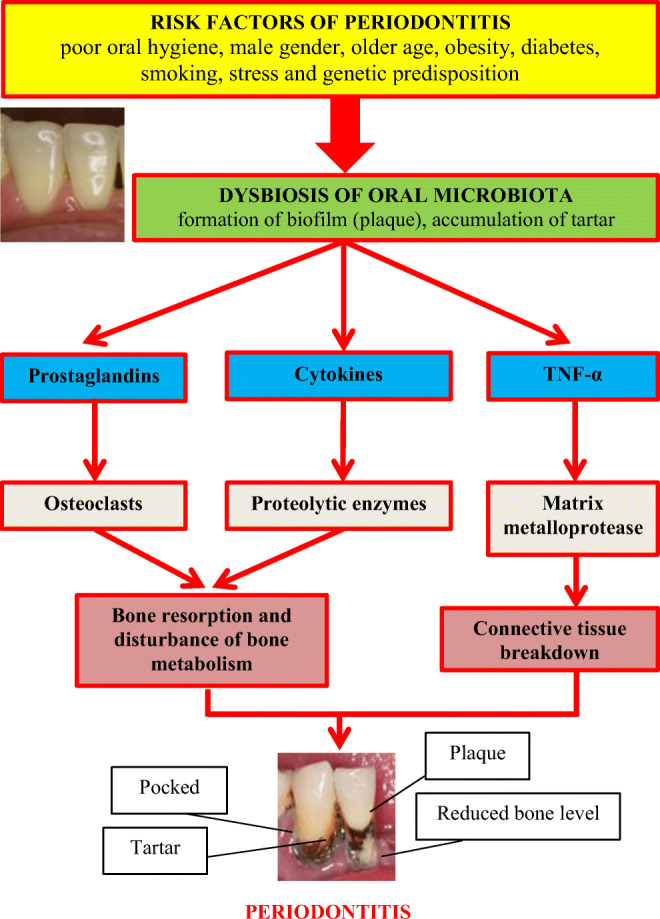
Etiopathogenesis of periodontitis. TNF-α, tumor necrosis factor α

More than 500 species of microorganisms, including bacteria, viruses, fungi, and protozoa, have been found in the oral cavity. Not all of the bacteria present are pathogenic. Periodontitis is associated with a mixed bacterial flora, with a predominance of anaerobic and microaerophilic bacteria. Bacteria causing periodontitis include, among others, *Porphyromonas gingivalis*, *Treponema denticola*, *Tannerella forsythia (Bacteroides forsythus)*, *Aggregatibacter actinomycetemcomitans*, *Prevotella intermedia*, *Streptococcus sanguis*, *Fusobacterium nucleatum*, etc. [11]. When hygienically neglected, bacteria colonize the cervical areas of the crowns of teeth, creating a plaque called biofilm, which is a specific ecological niche for them, protecting them from the effects of antiseptics and antibiotics. The risk factors for periodontitis include poor oral hygiene, male gender, older age, obesity, diabetes, smoking, stress, and genetic predisposition [11]. At this point, it is worth presenting the results of an interesting cross-sectional study conducted by Coelho et al. in which the impact of stress on the prevalence of periodontitis was assessed. The study included 621 people, of which 48.47% were classified as people burdened with stress (according to the Perceived Stress Scale). It was shown that, depending on the severity of stress, after adjusting for factors such as age, gender, education, current smoking habit, lung disease, and body mass index, the risk of periodontitis was increased by 15–36% (PR = 1.15; 95% CI 1.01–1.31 and PR = 1.36; 95% CI 1.01–1.83) compared to those without stress. Thus, common stressful situations may increase the risk of periodontitis [12•]. The main stages in the etiopathogenesis of periodontitis are shown in Fig. 3.

Prevalence of periodontitis in adults is significantly different in low- (28.7%), lower-middle- (10%), upper-middle- (42.5%), and high-income countries (43.7%) [13]. Severe forms of periodontal disease affect approximately 11% of the world’s population [14]. The incidence of periodontitis is higher in men (57% *versus* 39%) [15].

Periodontitis significantly increases the risk of cardiovascular diseases, which makes it a modifiable non-classical cardiovascular risk factor [16]. Arterial hypertension and periodontitis often coexist, especially in the elderly, men, cigarette smokers, overweight/obese people, diabetics, low socioeconomic status, and poor education [6]. Arterial hypertension occurs in 7–77% of patients with periodontitis (*versus* 4–70% in general population) [17••].

## Periodontitis and Blood Pressure

The influence of subgingival plaque bacteria on systolic and diastolic blood pressure was assessed by Desvarieux et al. In an observational study involving 653 people with no history of stroke or myocardial infarction, 4533 subgingival plaque samples were taken from the subjects (on average, 7 from each subject) and then analyzed for the presence of bacteria using the DNA-DNA hybridization technique. All analyses were adjusted for age, race/ethnicity, gender, education, body mass index (BMI), smoking, diabetes, low-density lipoprotein, and high-density lipoprotein cholesterol. It was shown that the mean systolic blood pressure increased in the terciles of the bacterial burden of the subgingival plaque from 136 to 138 mmHg and 143 mmHg in the 1st, 2nd, and 3rd terciles, respectively (*p* = 0.0004). Diastolic blood pressure also increased as follows: 77 mmHg, 79 mmHg, and 81 mmHg in the 1st, 2nd, and 3rd terciles of subgingival plaque burden, respectively (*p* <0.0001). It was found that systolic and diastolic blood pressure increased with increasing bacterial load and the subgingival plaque [18]. A study by Inoue et al. including 364 subjects (age 39.8±11.1 years) assessed the effect of periodontitis on blood pressure. It has been shown that patients with periodontitis had increased systolic and diastolic blood pressure both at baseline (128±2.1 mmHg *versus* 120.8±0.8 mmHg, respectively; *p* <0.001 and 129.2±2.3 mmHg *versus* 123.0±0.8 mmHg; *p* = 0.011 and 76±1.5 mmHg *versus* 71.2±0.6 mmHg; *p* = 0.003) and after 1-year observations (129.2±2.3 mmHg *versus* 123.0±0.8 mmHg, respectively; *p* = 0.011 and 80.5±1.7 mmHg *versus* 75.4±0.7 mmHg; *p* = 0.004) [19]. A study by Arowojolu et al. involving 100 people (age 51.0±13.6 years) also showed a significant relationship between periodontitis and increased systolic and diastolic blood pressure (*p* <0.000 and *p* <0.010, respectively) [20]. Similar results were obtained by Pietropaoli et al. in a study involving data of 7928 adults from NHANES III (National Health and Nutrition Examination Survey III). This study assessed the composition of the periodontal microbiota by determining antibodies against 21 oral pathogens. Among the assessed pathogens, three (*C. rectus*, *V. parvula*, and *P. melaninogenica*) were identified as more associated with the risk of arterial hypertension-associated oral pathogens (HOP), and the remaining were identified as NHOP (non-hypertension associated oral pathogens). HOP antibody development was consistently associated with a 3 mmHg and 2 mmHg increase in systolic and diastolic blood pressure, and with a 10 to 13% greater chance of high/uncontrolled blood pressure. Moreover, the presence of these antibodies was associated with more active periodontal disease and greater changes in the clinical parameters of periodontitis. Antibodies to *C. rectus* showed the strongest association with blood pressure. The researchers conclude that there is a relationship between the occurrence of periodontal pathogens and blood pressure [21].

It is worth emphasizing that the occurrence of periodontitis not only increases peripheral arterial pressure. In a study by Franek et al., including 99 people with advanced periodontitis (*n* = 50) or with no or moderate periodontitis (*n* = 49), differences with central blood pressure were assessed. It has been shown that advanced periodontitis was significantly associated with an increase in central systolic blood pressure (124±17 mmHg *versus* 116±15 mmHg, *p* <0.05) [22].

The most recent meta-analysis by Aguilera et al. covering 40 studies investigated, inter alia, the effect of periodontitis on systolic and diastolic blood pressure. It has been shown that patients with periodontitis had an increase in systolic and diastolic blood pressure of 4.49 mmHg (95% CI 2.88–6.11 mmHg; *p* < 0.00001) and 2.03 mmHg (95% CI 1.25–2.81 mmHg; *p* < 0.00001) compared to healthy subjects [17••]. Thus, observational studies have shown a positive correlation between periodontitis and increases in the blood pressure.

## Periodontitis and the Risk of Arterial Hypertension

The influence of periodontitis on the risk of arterial hypertension has been the subject of many clinical studies. A meta-analysis by Martin-Cabezas et al. involving 16 studies assessed the effect of periodontitis on the risk of arterial hypertension. It has been shown that moderate periodontitis increases the risk of arterial hypertension by 50% (OR 1.50; 95% CI 1.27–1.78) and severe periodontitis by 64% (OR 1.64; 95% CI 1.23–2.19). The authors of the meta-analysis indicate that the presence of common risk factors for periodontitis and arterial hypertension make it difficult to analyze this issue in greater detail. After taking into account the most important factors in common (age, gender), it was shown that the risk of arterial hypertension in people with periodontitis was increased by 16% (OR 1.16; 95% CI 1.07–1.26). Thus, the occurrence of periodontitis, especially of severe intensity, is associated with an increased risk of arterial hypertension [23•]. Zhao et al., in a retrospective cross-sectional study, assessed the impact of periodontitis on the risk of arterial hypertension in the Chinese population. The study included 3952 people aged 30–69 (including 2761 patients with arterial hypertension). It was shown that, depending on the confounding factors considered, the risk of arterial hypertension in patients with periodontitis was increased from 27 to 81% compared to healthy subjects. A subgroup analysis showed that the risk of developing arterial hypertension was highest in patients with periodontitis and up to 40 years of age [OR 1.694 (95% CI 1.196–2.398)], with BMI > 25 [OR 1.395 (95% CI 1.104–1.763)], with increased plasma LDL [OR 1.582 (95% CI 1.185–2.112)], or with an increased concentration of C-reactive protein in plasma [OR 1.783 (95% CI 0.298–10.671)]. Thus, periodontitis is significantly and positively correlated with an increased risk of hypertension in the Chinese population [24]. In another study, this time involving a representative group of Portuguese, Machado et al. also assessed the impact of periodontitis on the risk of arterial hypertension. The study included 1057 people (mean age 60.9±16.3; 532 were taking antihypertensive drugs), and the advancement of periodontitis was assessed on the basis of clinical attachment loss (CAL). It was shown that the risk of arterial hypertension depending on the stage of periodontitis (I, II, and III stages) was 1.72 (95% CI 1.10–2.57) in stage I, 2.60 (95% CI 1.82–3.72) in stage II, and 2.20 (95% CI 1.57–3.08) in stage III. All correlations were statistically significant, even after taking into account the effects of age, BMI, and smoking. It is worth emphasizing, however, that when the analysis was adjusted for age, the obtained relationships lost statistical significance. The analysis of the subgroup of subjects who did not take antihypertensive drugs showed a statistically significant effect of moderate periodontitis (stage II) on the risk of arterial hypertension [OR 2.60 (95% CI 1.61–4.21)], but after adjusting for age, this relationship lost statistical significance. Therefore, it was found that patients with periodontitis are characterized by an increased risk of arterial hypertension, but the age of the patient has a significant influence on this relationship [25]. Another cross-sectional study investigated the effect of periodontitis on the risk of prehypertension and arterial hypertension in Japanese students. The study included 2588 students who underwent a medical examination before starting their studies and before graduating (after 3 years). There was a significant relationship between the incidence of periodontitis and the risk of arterial hypertension (OR 2.74; 95% CI 1.19–6.29; *p* = 0.02), but no such association with the prehypertensive state (OR 0.93; 95% CI 0.51–1.70; *p* = 0.82). It was found that the occurrence of periodontitis is associated with an increased risk of hypertension among young people [26].

In order to more accurately determine the impact of periodontitis on the risk of arterial hypertension, Aguilera et al. in 2020 conducted the previously cited meta-analysis covering 40 studies. It was found that the odds ratio for the development of arterial hypertension in patients with periodontitis ranged in the analyzed studies between 0.90 and 4.20 depending on the stage of the disease and confounding factors. A statistically significant influence of periodontitis on the risk of arterial hypertension was demonstrated. This risk increased with the severity of periodontitis. The risk of arterial hypertension increases by 22–49% depending on the severity of the periodontitis (moderate to severe: OR 1.22; 95% CI 1.10–1.35 and severe: OR 1.49; 95% CI 1.09–2.05). The researchers conclude that periodontitis may be associated with an increased risk of arterial hypertension [17••].

A recent study by Pietropaoli et al., involving 8614 people over 30 years of age (NHANES III participants), assessed the relationship between periodontal inflamed surface area (PISA) and bleeding on probing (BoP) with the risk of arterial hypertension. PISA was treated as both continuous (mm^2^) and categorical (tertiles with cut-offs at 0, <37.6, and ≥37.6 mm^2^) variable. According to PISA tertiles, participants were identified as “not inflamed” (PISA = 0 mm^2^), “moderately inflamed” (0 <PISA <37.6 mm^2^), and “severely inflamed” (PISA ≥ 37.6 mm2). Compared to the absence of inflammation, severe PISA and BoP were associated with 43% (*p* <0.001) and 32% (*p* = 0.006) more likely to have high/uncontrolled BP (≥130 / 80 mmHg) and a higher systolic blood pressure by ≈4 (*p* <0.001) and 5 (*p* <0.001) mmHg. Thus, a significant relationship has been demonstrated between periodontitis and the risk of arterial hypertension [27••].

At this point, it is worth mentioning the results of the study by Taguchi et al., who assessed the impact of missing teeth on the risk of arterial hypertension in postmenopausal women. The study included 67 postmenopausal women with missing teeth and 31 without missing teeth. It was shown that missing dentition was associated with increased systolic and diastolic blood pressure (129.1±2.3 mmHg *versus* 121.6±2.9 mmHg and 78.9±1.5 mmHg *versus* 73.1±1.7). Arterial hypertension was more common in women with missing teeth (35.8% *versus* 12.9%). The odds ratio for arterial hypertension in women with missing teeth was 3.59 (95% CI 1.10–11.7) after adjusting for obesity, hypercholesterolaemia, and hypertriglyceridemia. Thus, arterial hypertension may be an important factor linking tooth loss and increased cardiovascular risk in postmenopausal women [28]. These results were confirmed in a study by Dar-Odeh et al. The study covered 1768 women aged 18–55 years. It was shown that missing teeth were significantly associated with the risk of arterial hypertension (*p* = 0.005). Interestingly, no correlation was found between the number of teeth with caries and the risk of arterial hypertension [29]. On the other hand, in the study by Darnaud et al., which included 102,330 people in a sample of people aged ≥65 years, no significant association was found between the oral variables (teeth loss, dental plaque, tartar, gingivitis) and the risk of arterial hypertension. In contrast, among subjects <65 years of age, an association was found between missing teeth (> 10 teeth), high levels of dental plaque, high horizontal tartar and gingivitis with the risk of arterial hypertension. The risk was 1.17 (95% CI 1.04–1.31), 1.90 (95% CI 1.55–2.33), 1.18 (95% CI 1.07–1.29), and 1.56 (95% CI 1.35–1.80). Thus, it was found that people under the age of 65 are more likely to develop arterial hypertension in the course of poor oral hygiene [30].

Interestingly, the potential role of oral microbiota dysbiosis in the pathogenesis of pregnancy hypertension and its complications has been suggested [31]. In a study by Pralhad et al., involving 200 pregnant women, the influence of periodontitis on the risk of pregnancy hypertension was analyzed. Prevalence of periodontitis was 65.5% and was significantly higher (*p* <0.0001) in females with pregnancy hypertension [RR 1.5 (95% CI 1.3–1.9)] [32]. A systematic review of the literature by Konopka and Zakrzewska, including 6 cohort studies, analyzed the effect of periodontitis on the risk of pre-eclampsia. Periodontitis has been shown to significantly increase the risk of pre-eclampsia (OR range from 2.4 to 5.89) [33]. A meta-analysis by Sgolastra et al. covering 15 studies showed that periodontitis increased the risk of pre-eclampsia [OR 2.17 (95% CI 1.38–3.41); *p* = 0.0008] [34].

Overall, periodontitis may contribute to a higher risk of pregnancy hypertension and its complications, such as pre-eclampsia.

Thus, observational studies have found a positive correlation between periodontitis and an increased risk of arterial hypertension. The factors modulating this relationship include the stage of periodontitis and the age of the respondents. In addition, tooth loss, dental plaque, tartar, and gingivitis significantly increase the risk of arterial hypertension.

## Periodontitis and the Control of Blood Pressure in Hypertensive Patients

From a clinical point of view, an important issue is the influence of periodontitis on blood pressure control in patients with arterial hypertension.

In a retrospective observational study, Pietropaoli et al. assessed the effect of periodontitis on blood pressure control in hypertensive patients. The study included 11753 adults aged 30 years and older with arterial hypertension and with or without periodontitis. There was a statistically significant relationship between the occurrence of periodontitis and higher systolic blood pressure (133.18 mmHg *versus* 130.12 mmHg; *p* <0.001). Moreover, periodontitis was significantly associated with about 20% higher risk of unsuccessful antihypertensive treatment compared with the absence of the disease. The authors conclude that the occurrence of periodontitis reduces the effectiveness of antihypertensive treatment [35••].

## Periodontitis Treatment and Blood Pressure—Interventional Studies

Taking into account the results of the above study, the impact of periodontitis treatment on blood pressure and arterial hypertension control seems to be important. So far, over a dozen intervention studies have been conducted with the participation of various groups of patients with periodontitis (Table 1) [17••].

**Table 1:** Intervention studies to assess the effect of periodontitis treatment on blood pressure

Author, year	Study design	Population characteristics	Intervention	Follow-up	Impact of periodontitis therapy on BP levels		
Seinost et al., 2005 [36]	Non-RCT	*N* =61 30 patients with severe periodontitis 31 healthy people—control group	Non-surgical treatment (2 sessions) and pharmacological methods (antibiotic therapy; 7 days)	3 months	No changes in blood pressure. There was no statistically significant change in SBP (*p* = 0.68) and DBP (*p* = 0.2) at follow-up		
D’Aiuto et al., 2006 [37]	RCT	40 subjects with chronic periodontitis - Standard periodontal therapy (SPT)= 20 subjects - Intensive therapy (IPT)= 20 subjects	SPT—non-surgical treatment (1 session) IPT—non-surgical treatment (1 session) and LDA (locally delivered antimicrobials): minocycline microspheres	1, 2, 6 months	Transient reduction in systolic blood pressure in IPT that returned to baseline after 6 months (135±14mmHg → 129±17mmHg; *p* = 0.04) No effect on diastolic blood pressure		
Tonetti et al., 2007 [38]	RCT	120 subjects with severe periodontitis - 59 intervention - 61 control	Intervention group: oral hygiene instruction (OHI), single session of NST + LDA (microspheres of minocycline) and extraction of hopeless teeth Control group: OHI, single session of supra-gingival scaling and polishing	Days 1, 7, 30, 60,180	Mean difference in SBP/DBP in the test group *versus* control (day 1). SBP= 4.60 mmHg (1.07–8.13); *p*=0.01, DBP= 2.95 mmHg (0.42–5.49); *p*=0.02 No effect after 6 months		
Higashi et al., 2008 [39]	RCT	Protocol 1: *n*=52 Groups: - 32 subjects with periodontitis randomly allocated to periodontal therapy (16) or no intervention (16) - 20 healthy controls (periodontally and systemically) Protocol 2: *n*=64 Groups: - 26 subjects with periodontitis randomly allocated to periodontal therapy (17) or no intervention (9) - 38 periodontally-healthy individuals The study included people with arterial hypertension	Protocol 1 + 2: Periodontal therapy group: OHI, NST and 4–7 days of systemic antibiotic and mouthwash for 24 weeks - Control group, and no intervention group did not receive periodontal therapy	Protocol 1 and 2: 24 weeks	Slight decrease in blood pressure		
					Protocol 1		
						Before	After
					SBP [mmHg]	115.1±10.9	114.6±10.4
					DBP [mmHg]	66.1±7.4	67.4±7.9
					Protocol 2		
						Before	After
					SBP [mmHg]	140.1±20.3	141.3±20.3
					DBP [mmHg]	89.2±14.1	88.7±13.7
					However, the results obtained in both protocols were not statistically significant		
Higashi et al., 2009 [40]	RCT	*N*=101 subjects with coronary artery disease randomly allocated to treatment or control groups: - 48 subjects with chronic periodontitis - 53 controls (no periodontitis)	- Periodontitis subjects randomly allocated to periodontal therapy that was NST and 4 to 7 days of antibiotic and mouthwash for 24 weeks (24) or no intervention (24) - Control group: no periodontitis treatment both groups: intra-arterial infusion of acetylcholine and to sodium nitroprusside before periodontal therapy in 48 subjects with periodontitis and 53 control subjects, and in 24 patients who were treated periodontitis and 24 untreated subjects before and after 24 weeks of follow-up	24 weeks	Slight decrease in blood pressure		
					Study group		
						Before	After
					SBP [mmHg]	141.3±20.2	140.3±19.3
					DBP [mmHg]	82.5±13.3	80.7±12.9
					Control group		
						Before	After
					SBP [mmHg]	141.3±19.8	140.8±19.1
					DBP [mmHg]	82.3±12.7	81.9±12.1
					However, the results obtained in both protocols were not statistically significant		
Taylor et al., 2010 [41]	RCT	*N*=125 subjects with periodontitis Groups: - Randomly allocated to - Test (61) - Control group (64)	- Test OHI, extractions of hopeless teeth, NST - control dental extractions to alleviate pain No periodontitis treatment	3 months	No effect on blood pressure (data obtained from authors by Aguilera E. et al. [17••])		
Graziani et al., 2010 [42]	Non-RCT	*N*=14 subjects with severe chronic periodontitis Groups: only intervention group	OHI, scaling and root planing (SRP) in 2 visits within 24 hours, Widman flap procedure 180 days after completion of NST	Days 1, 7, 30, 90, 180, 181, 187, 200, 201, 207, 270	No effect on blood pressure		
López et al., 2012 [43]	RCT	N=165 subjects with periodontitis and metabolic syndrome Groups: - Experimental treatment group = 82 - Control group = 83	- Experimental group: received plaque control and NST plus amoxicillin and metronidazole - Control group: received plaque control instructions, supragingival scaling, and two placebos	12 months	Not statistically significant effect on blood pressure		
Vidal et al., 2013 [44]	Non-RCT	*N*=26 subjects with chronic periodontitis and refractory hypertension. Groups: only intervention group	-OHI, NST no time limit Blood pressure was assessed using an ambulatory blood pressure monitoring (ABPM)	6 months	Statistically significant reduction in systolic and diastolic blood pressure by 12.5 mmHg and 10 mmHg, respectively		
Hada et al., 2015 [45]	RCT	N=70 subjects with coronary heart disease and periodontitis Groups: - Experimental: 35 - Control: 35	- Experimental group—non-surgical periodontal treatment in the form of scaling and root planing - Control group—no periodontitis therapy	1, 3, 6 months	Highly statistically significant reduction was observed in systolic BP Systolic and diastolic blood pressure: baseline: 130.80±22.58 mmHg and 82.24±11.68 mmHg after 6 months: 123.70±15.72 mmHg and 80.07±9.12 mmHg		
Houcken et al., 2016 [46]	Pilot study	*N*=45 subjects with periodontitis out of 109 that participated in the cross sectional study groups: only intervention group	Treatment: OHI + NST in 2 sessions. Twenty patients (randomly assigned) received a systemic antibiotic therapy adjunctive to the scaling and root planning (combination of Amoxicillin 375mg t.i.d. and Metronidazole 500mg t.i.d. for 7 days)	6 months	Decrease of blood pressure Statistically significant reduction in systolic blood pressure (119.8±14.6 mmHg is 116.9±15.1 mmHg; p=0.04). Reduction in diastolic blood pressure (74.9±11.8 mmHg to 73.1±10.6 mmHg; *p*=0.05)		
Zhou et al., 2017 [47]	RCT	N=95 subjects with periodontitis, pre-hypertension Groups: Randomly assigned to test and control groups: - Intensive treatment group: 48 - Control treatment group: 47	Intensive treatment group: OHI, NST+ locally delivered minocycline hydrochloride ointment (once a week for 4 weeks). Additionally, teeth that could not be saved were extracted - Control treatment group only supragingival ultrasonic scaling and polishing at baseline	6 months	Significant reduction in systolic and diastolic blood pressure in the intensive care group compared to the control group: (systolic blood pressure/diastolic blood pressure: 12.57 mmHg/9.65 mmHg, 95% CI 10.45–14.69 mmHg and 06–12.24 mmHg; *p* < 0.05)		
D’Aiuto et al., 2018 [48]	RCT	*N*=264 subjects with moderate-severe periodontitis and at least 15 teeth and diabetes type 2 Groups: - Intensive therapy: 133 - Control therapy: 131	Intensive therapy—initial single session of whole mouth scaling of the root surfaces. 2 months after patients with (plaque scores of ≤ 20%) and at least one PPD⩾6 mm had periodontal surgical therapy followed by repeated scaling every 3 months. Control therapy - cleaning and polishing the part of the tooth that is visible above the gingiva of all dentition at the same time-points as the IPT group (after baseline and at 2, 6, 9, and 12 months after the completion of the first session of periodontal therapy)	12 months	Blood pressure change was not statistically significant in both groups		
Czesnikiewicz-Guzik et al., 2019 [49••]	RCT	*N*=101 subjects with arterial hypertension and periodontitis Groups: - Intensive therapy: 50 - Control therapy: 31	Intensive therapy—sub- and supragingival scaling/chlorhexidine Control therapy—supragingival scaling Blood pressure was assessed using an ambulatory blood pressure monitoring (ABPM)	2 months	Statistically significant reduction in blood pressure Systolic blood pressure: −11.1 mmHg (95% CI 6.5–15.8 mmHg) after 2 months of intensive therapy (*p* <0.01) Diastolic blood pressure: −8.3 mmHg (95% CI 4.0–12.6 mmHg) after 2 months of intensive therapy (*p* <0.01)		

Interventional studies showed inconsistent results regarding the effect of periodontitis treatment on blood pressure. Nine studies showed no effect of periodontitis treatment on blood pressure. On the other hand, 5 more recent clinical trials showed a reduction in blood pressure in the treated patients. This effect was diversified and reached even 12 mmHg and 10 mmHg for systolic and diastolic blood pressure, respectively. It seems that the method of its measurement is important in assessing the effectiveness of periodontitis treatment on arterial pressure. In studies where measurements were made using the 24-h blood pressure monitoring (ABPM) method, the greatest reduction in systolic and diastolic blood pressure was demonstrated. The observed differences in blood pressure reduction in patients after periodontitis treatment may also result from genetic predisposition. The study by Czesnikiewicz-Guzik et al. showed that single nucleotide polymorphisms (SNPs) in genes loci associated with periodontitis were also significantly associated with increased blood pressure [49••].

Therefore, it seems that the treatment of periodontitis may be an important method of preventing and controlling arterial hypertension.

In the previously cited systematic review by Konopka and Zakrzewska, 3 randomized clinical trials were analyzed in which the impact of scaling and root planning on the risk of pre-eclampsia was assessed. Treatment of inflammation has not been shown to reduce the risk of pre-eclampsia [33].

## Pathophysiology of Arterial Hypertension in Patients with Periodontitis

**Fig. 4:**
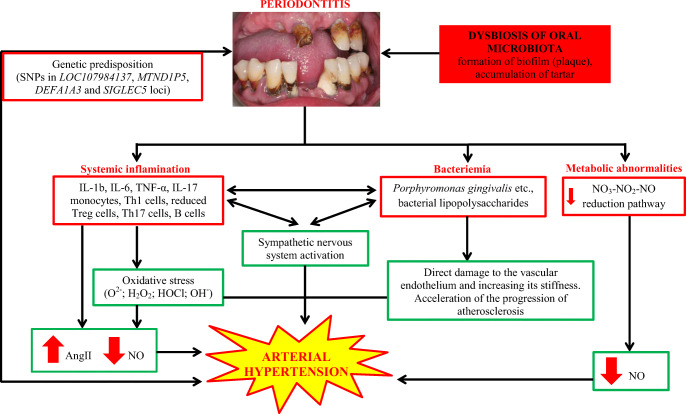
Pathogenesis of arterial hypertension in periodontitis

The pathogenesis of arterial hypertension in patients with periodontitis is complex and not fully understood; therefore, some authors suggest the term “dental hypertension” to emphasize the importance of this problem [50–53]. It seems that the main pathomechanism responsible for increasing blood pressure in patients with periodontitis is systemic inflammation and secondary damage to the vascular endothelium [50–53]. As indicated by Del Pinto et al., the estimated periodontium area is equal to the area of the hand. The influence of local inflammation of such a large extent occurring during generalized periodontitis may significantly contribute to systemic inflammation [6]. The pathogenesis of arterial hypertension in periodontitis is presented in Fig. 4.

The most important factor involved in the pathogenesis of arterial hypertension in patients with periodontitis is dysbiosis of the oral microbiota. As previously described (Fig. 1), oral bacteria participate in the production of nitric oxide. Pathological changes in the composition of oral bacteria in patients with periodontitis may lead to a reduction in the production of nitric oxide, which in turn may contribute, apart from other mechanisms described, to an increase in blood pressure [7–9].

Periodontitis is the cause of a systemic inflammatory process mediated by C-reactive protein, interleukin 1b (IL-1b), interleukin 6 (IL-6), and tumor necrosis factor alpha (TNF-α), among others [54]. These factors can directly affect the vascular endothelium, leading to impairment of its vasodilatory function [17••]. It was shown that the treatment of periodontitis improved the function of the vascular endothelium both in people with comorbidities such as diabetes and in people without them [38, 48]. Interestingly, it was also found that the immune response to a common periodontal pathogen *Porphyromonas gingivalis* led directly to an increase in blood pressure, vasculitis, and impaired vascular endothelial function [49••]. Another possibility is that periodontal inflammation increases the chemotactic activity of T lymphocytes, B lymphocytes, and monocytes, leading to vascular dysfunction, increased progression of atherosclerosis, and increased blood pressure [55, 56]. Studies in recent years have shown the central role of T cells in the development of arterial hypertension [57–60]. Specifically, following hypertensive stimuli, activated T cells accumulate in the perivascular tissue, where they release cytokines (i.e., TNF-α, IL-6, IL-17) that, in turn, contribute to the development of high blood pressure [57–60]. Moreover, a special role of certain lymphocyte subclasses in the pathogenesis of hypertension has been demonstrated. CD8 T cell senescence is an important feature of arterial hypertension [61]. In the previously cited study by Czesnikiewicz-Guzik et al., it was shown that the treatment of periodontitis led to a decrease in the percentage of CD8 and CD28 null CD57+ cells. Additionally, in this study, after the treatment of periodontitis, a decrease in the concentration of interferon gamma (INF-γ), IL-17A, TNF-α, and IL-6 in the blood was demonstrated [49••]. Moreover, Th17 lymphocytes play an important role in the impairment of the vasodilatory function of the endothelium, which by secreting IL-17 lead to an increase in the production of superoxide, which in turn reduce the nitric oxide-dependent vasodilation [62]. Interestingly, the dysbiosis-dependent increase in the percentage of Th17 lymphocytes also appears to be of importance in the pathogenesis of periodontitis. It has been shown that people with natural Th17 deficiency suffered from periodontitis less frequently [63, 64]. Another subset of T cells of importance in both are regulatory T cells (Treg). Unlike Th17, Tregs have a protective effect in arterial hypertension by angiotensin II antagonism and reduction circulating activated T cells [65]. Moreover, Tregs attenuate the severity of periodontitis by increasing the secretion of anti-inflammatory cytokines such as IL-10 and TGF-β [66]. In the course of periodontitis, their percentage may decrease, intensifying the mechanisms leading to the development of arterial hypertension [6, 17••]. The genetic polymorphisms mentioned above may also contribute to the pathogenesis of arterial hypertension in the course of periodontitis [49••].

As mentioned, periodontitis may exacerbate the progression of atherosclerosis, leading to impaired vascular endothelial function. Periodontal pathogens may directly increase the progression of atherosclerotic lesions. Haraszthy et al. evaluated the composition of atherosclerotic plaques in the carotid arteries, using 50 samples of biological material collected from patients during the endarterectomy procedure. Interestingly, it was shown that 44% of the 50 atheromas were positive for at least one of the target periodontal pathogens. Thirty percent of the surgical specimens were positive for *B. forsythus*, 26% were positive for *Porphyromonas gingivalis*, 18% were positive for *A. actinomycetemcomitans*, and 14% were positive for *P. intermedia*. It was found that periodontal pathogens present in the atherosclerotic plaque may be involved in the progression of atherosclerotic lesions [67]. The previously cited study by Arowojolu et al. showed a significant positive relationship between the occurrence of periodontitis and mean carotid artery intima media thickness and oral hygiene index (*p* <0.012) [20]. In addition, a recent clinical study involving 2888 participants has shown that periodontitis increases arterial stiffness as assessed by the cardio-ankle vascular index (CAVI) method [68].

## Arterial Hypertension and Periodontitis: Common Genetic Predisposition Factors

The genome-wide association study (GWAS) demonstrated that SNPs, in LOC107984137 (rs729876), MTND1P5 (rs16870060), DEFA1A3 (rs2738058), and SIGLEC5 (rs4284742) loci, was associated with periodontitis [69, 70]. In the study by Czesnikiewicz-Guzik et al., using Mendelian randomization, it was shown that all four studied SNPs showed also concordant effect direction, i.e., the same alleles were associated with both increased risk for periodontitis and increased level of blood pressure (Fig. 4.) [49••]. The results of this study explain one of the possible causes of the frequent coexistence of periodontitis and arterial hypertension.

## Use of Mouthwash and the Risk of Arterial Hypertension

**Fig. 5:**
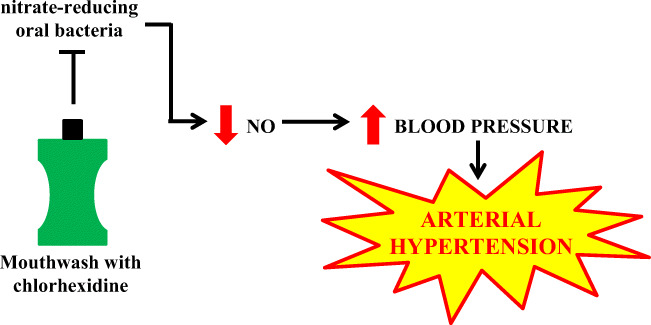
The pathogenesis of arterial hypertension induced by the use of mouthwashes

The use of chlorhexidine mouthwashes may reduce the bacterial blood pressure lowering effects associated with nitrates. An imbalance in the oral cavity reducing the microflora is associated with a decrease in NO, favoring endothelial dysfunction and an increased cardiovascular risk (Fig. 5.) [7–9].

These pathophysiological links were confirmed in a recent study by Joshipura et al. involving 540 participants. This study looked at the effects of the use of an over-the-counter mouthwash on the risk of arterial hypertension. It has been shown that people who used mouthwash twice daily or more often had a higher incidence of arterial hypertension compared to less frequent users (incidence rate 1.85; 95% CI 1.17–2.94) and compared with non-users (IRR = 2.17; 95% CI 1.27–3.71). The use of the mouthwash less than 6 times a week and once a day compared to non-use was not significantly associated with the risk of arterial hypertension (IRR 1.58; 95% CI 0.78–3.18 and IRR 1.30, 95% CI 0.70–2.41, respectively). The study authors conclude that frequent, regular use of over-the-counter mouthwashes was associated with an increased risk of hypertension, regardless of the major risk factors for hypertension and several other potential confounding factors [71••]. It is worth mentioning that the same researchers showed that too frequent use of mouthwash with chlorhexidine increased the risk of type 2 diabetes [72].

In an interesting study by Tribble et al., involving 27 people free of oral disease, the effect of the use of mouthwash with chlorhexidine on blood pressure and the composition of the oral microbiota was assessed. The twice-daily chlorhexidine usage was associated with a significant increase in systolic blood pressure after 1 week of use and recovery from use resulted in an enrichment in nitrate-reducing bacteria on the tongue [73].

In summary, too frequent use of mouthwash with chlorhexidine reduces the level of nitrate-reducing bacteria in the oral cavity, which leads to an increase in blood pressure. Thus, it appears that using a mouthwash once a day should be safe and not increase the risk of arterial hypertension.

People who have been recommended by the dentist to use an antibacterial mouthwash should use it less than 6 times a week. The potential benefits and risks of using such fluids in people with hypertension should be weighed. In other people with no indications for the use of such fluids, oral hygiene is based on brushing teeth twice a day and following a healthy diet.

## Conclusions

Periodontitis is a disease of social importance. It occurs in about 50% of the world’s population. The risk factors for periodontitis include poor oral hygiene, male gender, older age, obesity, diabetes, smoking, stress, and genetic predisposition. Arterial hypertension occurs in 7–77% of patients with periodontitis and is still neglected area of research, though the periodontal diseases are being connected often with coronary syndromes or heart failure, also in our own studies published before [74–77]. Periodontitis can lead to an increase in blood pressure (meta-analysis of observational studies). Depending on the severity, periodontitis increases the risk of developing arterial hypertension by 22–49% (meta-analysis of observational studies). Periodontitis was significantly associated with about 20% higher risk of unsuccessful antihypertensive treatment (observational studies). Treatment of periodontitis may reduce systolic and diastolic blood pressure by 12 mmHg and 10 mmHg, respectively (some interventional studies). The pathogenesis of arterial hypertension in periodontitis is complex. The most important factor involved in the pathogenesis of arterial hypertension in periodontitis is dysbiosis of the oral microbiota. The important role of the immune system, impaired vascular endothelial functions and perhaps a direct acceleration of the progression of atherosclerosis are emphasized. Periodontitis could be considered the modifiable non-classical risk factor for arterial hypertension. Taking care of oral hygiene and treating periodontitis should be a method of preventing arterial hypertension and a method improving the effectiveness of antihypertensive treatment. In order to maintain oral hygiene, mouthwash should not be used too often, as such use interferes with nitric oxide homeostasis and increases the risk of arterial hypertension.

**Fig. 6:**
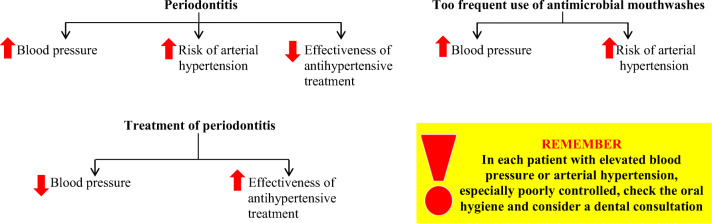
Information for the general practitioner on the relationship between periodontitis and arterial hypertension

Summary of information for a general practitioner on the relationship between periodontitis and arterial hypertension is presented in Fig. 6.
